# Does the US medical physics community have the optimal certification model for the next decade?

**DOI:** 10.1002/acm2.12781

**Published:** 2019-11-27

**Authors:** Michael Mills

Most of our medical physicists in training are longing for the day when they receive notification that they have passed the American Board of Radiology Certification examination in one of the subfields of medical physics practice. This Certificate gives the holder the needed qualification to apply for positions requiring such a certification and designates the candidate as a Qualified Medical Physicist (QMP). Given its significant importance in our industry, it is desirable periodically to review the ABR Board Certification structure to assure it is meeting the needs of the medical physics community and the patients served by that community.

One drawback of the current structure is that the medical physics trainee is required to select a specialty area very early in their career as a medical physicist. It is difficult enough to obtain and maintain certification in one specialty; maintaining certification in two or three areas is both very onerous and rare. However, if a student wishes to preserve the option of changing their specialty practice or of practicing more than one specialty, there seems little option but to plan for this early in their career.

I am noticing that a great many articles submitted to the JACMP are broadly conceived. Many radiation oncology physics papers contain substantial imaging science and clinical practice. Many imaging articles contain radiation oncology imaging and clinical concepts. There are Associate Editors in the JACMP who handle both radiation oncology and imaging manuscripts. Radiation oncology physicists must support the imaging equipment in the department in many if not most institutions, so is the strong division of practice between radiation oncology and imaging at least somewhat artificial?

A review of physician ABR certifications and subspecialties within radiology reveals a well‐thought listing of professional certifications with specific additional competencies in subspecialty divisions. Most of these subspecialty competencies require an additional year of training within the subspecialty. There is no counterpart to this respecting medical physics clinical training and certifications.

Consider these legacy certifications awarded by the ABR (https://www.theabr.org/about/certificate-history):

Legacy Certificates

The following certificates, though still valid, are no longer offered by the American Board of Radiology. They were issued during the years indicated. Please note that these certificate names must be permanently updated to the current name(s) to be eligible for Maintenance of Certification.

Radiologic Physics (now Medical Physics), 1947–1997

X‐Ray and Radium Physics, 1947–1960

Roentgen Ray & Gamma Ray Physics, 1961–1975

Therapeutic and Diagnostic Medical Physics, 1973–1997

Diagnostic and Nuclear Medical Physics, 1976‐1998

Therapeutic and Nuclear Medical Physics, 1976–1992

Radiologic Physics was certified by the ABR for 50 years, and all of these certificates are still active. Additionally, one could get certified individually in two or three areas, if desired, in order to exhibit certification in each of their practice areas. However, given the difficulty of maintaining multiple certifications, some institutions hire a radiation oncology medical physicist to manage all of their imaging equipment (radiation oncology and radiology) on a full‐time basis, thus effectively having the physicist practicing imaging physics under a radiation oncology physics certificate.

All of these factors lead me to make the following observations and offer a suggestion: Many medical physicists practice broadly, and often without specialty certification in some components of their practice. This is often overlooked. It is possible we have made a collective error in judgment when as a community we engaged in a headlong rush into specialization a generation ago? Would we have been better off continuing to examine for primary certification only in Medical Physics? Could we have added subspecialties in radiation oncology physics, imaging physics and nuclear medical physics? Would we have the flexibility to add (and perhaps remove at some point) more subspecialties presently and in the future? Would initial certification as a medical physicist and maintenance of certification be of better service to the community and to our patients if it were conceptually broader and not limited to narrowly conceived specialty questions?

Conflated with all of these facts is the overall evolution of our service parameters. New and large subgroups of practice have emerged and are emerging, such as brachytherapy physics, radiosurgery physics, radiation oncology particle physics, mammography physics, MRI physics and others. New technologies such as machine learning, radiomics, artificial intelligence, remote provision of services, and other workforce issues further complicate the medical physics specialty landscape.

I further submit that our leadership in the AAPM, CAMPEP and the ABR should seriously think about what is best for the profession and ask ourselves whether we have the structure that best serves the interests of our membership and the patients they serve. (Radiologic) Medical Physics was offered by the ABR as a physics certification as late as 1997. Maybe it should have never gone away.

In full disclosure, my own career included ABR certification in all three areas, not as a Radiologic Physicist, but rather I took each of the three certifications individually. I believe this broad practice training and certification experience helped me greatly in my career, and I wish this type of pathway were more accessible for physicists today. While I elected not to participate in Maintenance of Certification with the ABR, I do maintain ABMP certification in Radiation Oncology Physics.

